# Validation of dynamic [^18^F]FE-PE2I PET for estimation of relative regional cerebral blood flow: a comparison with [^15^O]H_2_O PET

**DOI:** 10.1186/s13550-022-00941-8

**Published:** 2022-11-17

**Authors:** Susanna Jakobson Mo, Jan Axelsson, Lars Stiernman, Katrine Riklund

**Affiliations:** 1 Department of Radiation Sciences, Diagnostic Radiology, Umeå University, Umeå, Sweden; 2 Umeå Centre for Functional Brain Imaging (UFBI), Umeå University, Umeå, Sweden; 3 Department of Radiation Sciences, Radiation Physics, Umeå University, Umeå, Sweden; 4 Dept. of Integrative Medical Biology, Umeå University, Umeå, Sweden

**Keywords:** Positron emission tomography, ^18^F FE-PE2, ^15^O H_2_O, Cerebral blood flow, Cerebral perfusion, Parkinsonism, Parkinsonian syndromes

## Abstract

**Background:**

Dopamine transporter (DAT) imaging is used in the diagnostic work-up in suspected parkinsonian syndromes and dementia with Lewy bodies but cannot differentiate between these syndromes, and an extra brain imaging examination of the regional cerebral blood flow (rCBF) or glucose metabolism is often needed for differential diagnosis. The requirement of two different imaging examinations is resource-consuming and inconvenient for the patients. Therefore, imaging of both cortical blood flow and DAT imaging with the same radiotracer would be more convenient and cost-effective. The aim of this study was to test whether relative regional cerebral blood flow (rCBF_R_) can be measured with the DAT-specific positron emission tomography (PET) tracer [^18^F]FE-PE2I (FE-PE2I), by validation with cerebral perfusion measured with [^15^O]H_2_O PET (H_2_O).

**Methods:**

The rCBF_R_ was quantified by kinetic modeling for FE-PE2I (*R1*) and H_2_O (*F*). The *R1* was calculated using the simplified reference tissue model, and *F* was calculated with a modified Koopman double-integration method. The linear relationship and intraclass correlation (ICC) between *R1* and *F* were tested in image data derived from 29 patients with recent onset parkinsonism and 30 healthy controls.

**Results:**

There was a strong linear correlation across all subjects between *R1* and *F* in the frontal, parietal, temporal, cingulate and occipital cortex as well as in the striatum (*r*  ≥  0.731–0.905, *p * < 0.001) with a good-to-excellent ICC, ranging from 0.727 to 0.943 (*p* < 0.001).

**Conclusions:**

Our results suggest that FE-PE2I may be used as a proxy for cerebral perfusion, thus potentially serving as a radiotracer for assessment of both DAT availability and rCBF_R_ in one single dynamic scan. This could be valuable in the differential diagnosis of parkinsonian syndromes.

*Trial registration*: EUDRA-CT 2015-003045-26. Registered 23 October 2015 https://www.clinicaltrialsregister.eu/ctr-search/search?query=2015-003045-26

**Supplementary Information:**

The online version contains supplementary material available at 10.1186/s13550-022-00941-8.

## Background

Dopamine transporter (DAT) imaging is used to detect dopaminergic deficit in striatum to support the diagnosis of Parkinson’s disease (PD) and atypical parkinsonian syndromes, i.e., progressive supranuclear palsy (PSP), multisystem atrophy (MSA), corticobasal degeneration (CBD), and dementias with Lewy bodies. To date, only a few studies have shown a differential diagnostic potential for these disorders using DAT positron emission tomography (PET) imaging alone, based on striatal subregional DAT binding [1–3]. Consequently, in practice, supplementary cerebral perfusion or glucose metabolism brain imaging is often required to support the differential diagnosis between different parkinsonian conditions and syndromes [4–10]. The necessity of two different PET examinations with different tracers is costly and resource-intensive in the diagnostic work-up. Therefore, the idea of using early and late image data collected within one single tracer PET examination for assessment of both cerebral perfusion (as a measure of cortical function) and the specific DAT binding (as a biomarker for presynaptic dopaminergic function) has been successfully validated for some DAT tracers in studies compared with radiopharmaceuticals for perfusion or glucose metabolism [11–14].

Several published studies have investigated different aspects of DAT imaging with the relatively new, highly selective DAT tracer [^18^F]-(E)-N-(3-iodoprop-2-enyl)- 2β-carbofluoroethoxy-3β-(4'-methyl-phenyl) nortropane (FE-PE2I) [15] in parkinsonian disease and in healthy individuals [16–24]. Similar to ^11^C-PE2I, the fluorinated variant, i.e., FE-PE2I, allows for high-quality, quantifiable imaging of DAT activity in both the striatum and the substantia nigra [25]. However, the compounds differ, with FE-PE2I having faster kinetics and less metabolites, allowing for a shorter scanning protocol, less complicated quantification, and the longer half-life of ^18^F is more practical for clinical use [26, 27]. To our knowledge, regional cerebral blood flow (rCBF) with FE-PE2I has not yet been evaluated.

Perfusion can be measured directly with ^15^O-labeled H_2_O and is considered gold standard if arterial blood sampling is used as input to pharmacokinetic modeling of the PET time–activity curve. A relative regional cerebral blood flow (rCBF_R_) measure can be estimated using reference tissue models when arterial sampling is not available [28]. Measures of both receptor binding, quantified as binding potential (BP) and *R1*, can be estimated at the same time, using the simplified reference tissue model (SRTM) [29, 30].

The aim of this study was to assess the correspondence between rCBF_R_ measured with dynamic FE-PE2I (*R1*) and ^15^O-labeled H_2_O (*F*). The hypothesis is that FE-PE2I has similar mobility into tissue as water, signified by *R1* correlating highly with both absolute and relative rCBF measured with H_2_O.

A secondary aim was to assess measurable differences in rCBF_R_, measured with *R1* and *F,* between healthy controls (HC) and patients with recent onset parkinsonism.

## Methods

This study presents parts of the secondary end-points of a non-commercial clinical trial evaluating the diagnostic potential of FE-PE2I in early-stage idiopathic parkinsonism, the PEARL-PD study ([^18^F]FE-PE2I PET/CT study of Dopamine Transporters in Early Parkinsonian disease, Eudra-CT no.: 2015–003045-26) finalized in June 2020.

**Fig. 1:**
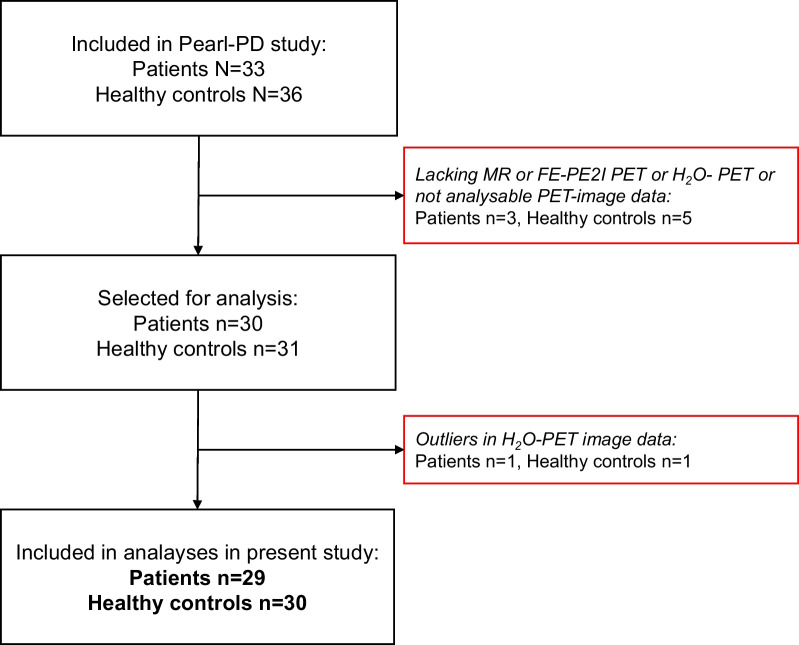
STARD diagram showing the selection of data for this study

FE-PE2I- and H_2_O image data were collected from the PEARL-PD study. Thirty patients with recent onset of idiopathic Parkinsonism according to established clinical criteria [31] without signs of cognitive impairment and 31 HC within the same age range had both an FE-PE2I and an H_2_O. Two participants, one HC and one patient, were excluded from the analyses as their estimated *F* was ≥ 2.5 standard deviations (SD) from the mean. Thus, the results presented in this paper are based on 29 patients and 30 HC. In Fig. 1, the selection of patients and HC for analyses in this paper is illustrated. Twenty-five patients had a reduced BP of FE-PE2I in the striatum (i.e., reduced DAT activity). Four patients had normal striatal DAT activity, of whom three had a non-degenerative parkinsonian condition (non-IPS) and one with an atypical parkinsonian syndrome. An overview of the basic characteristics of the 59 participants is given in Table 1.

**Table 1:** Basic statistics

		Healthy volunteers	Patients (follow-up diagnoses)		Total
			IPS or DLB	Non-IPS	
Sex	Women, N	16	9	2	26
	Men, N	14	17	1	32
Age at inclusion, years	Median	70.0	68.8	71.6	69.3
	Mean	70.3	68.3	68.4	69.3
	SD	4.7	7.6	12.3	6.5
Symptoms’ duration, years	Median	–	0.7	1.0	0.7
	Mean	–	1.2	1.3	1.2
	SD	–	1.2	0.6	1.1
Baseline UPDRS III	Median	0.5	23	17	7
	Mean	1	23	16	11
	SD	2	7	6	12
Baseline MMSE	Median	30	29	30	29
	Mean	29	29	30	29
	SD	1	1	1	1

*IPS* Idiopathic parkinsonian syndromes, i.e., degenerative parkinsonian syndrome, such as Parkinson’s disease or atypical parkinsonian syndrome. *DLB* Dementia with Lewy bodies

*Non-IPS* Patients with non-degenerative parkinsonism, e.g., vascular parkinsonism or other condition with tremor or Parkinson-like symptoms

The PET scans with FE-PE2I and H_2_O were performed on the same day in the majority of cases. In ten cases, the H_2_O was done separately within a median interval of 14 days (range 3–47 days) from the FE-PE2I due to technical issues. The H_2_O served as the standard for cerebral perfusion [32–34]. In the study protocol, a 3 T magnetic resonance (MR) of the brain was also included. The PET imaging was done at baseline (after inclusion), before start of any pharmacological treatment.

Both patients and HC were clinically examined both at baseline and at follow-up using neurological, cognitive, and olfactory testing. Unbiased by the imaging results, a tentative clinical diagnosis was set at baseline, based on established clinical criteria, and re-evaluated 2 years later. Four of the patients included in this study had no signs of dopaminergic deficit, so-called subjects without evidence of dopaminergic deficit (SWEDD) at the baseline FE-PE2I. Three of these patients were clinically diagnosed with non-idiopathic parkinsonism (e.g., vascular parkinsonism) and one fulfilled the clinical criteria for an atypical parkinsonian syndrome at the clinical re-evaluation at follow-up 2 years later.

### Radiosynthesis

The PET radiopharmaceuticals were synthesized on-site at the PET center at the University Hospital of Umeå. [^18^F]FE-PE2I was synthesized according to previously described methods [35]. The production of [^15^O]H_2_O was performed with a Hidex Radiowater Generator system (Hidex Oy, Åbo, Finland).

### Imaging protocol

First, a 6-min dynamic H_2_O was acquired, followed by a short rest (median 17 min) waiting for ^15^O radioactive decay, after which a 75-min dynamic FE-PE2I was performed. Both acquisitions were performed in the same session including a low-dose computed tomography (CT) for attenuation correction on a Discovery 690 PET/CT (General Electric, Milwaukee, WI),

#### [^15^O]H_2_O PET

The PET scanning commenced at the start of a bolus intravenous infusion of 800 MBq H_2_O, delivered by the Hidex generator. Total scanning time was 370 s (14 × 5 s, 3 × 10 s, 3 × 20 s, 7 × 30 s).

#### [^18^F]FE-PE2I

The FE-PE2I image protocol was described in a previous publication [19]. In short, a 75-min dynamic PET acquisition (9 × 20 s, 3 × 60 s, 5 × 180 s, 9 × 360 s) commenced at the intravenous bolus injection of 2.86 MBq/kg [^18^F]FE-PE2I.

### Image reconstruction

PET series were reconstructed using the built-in “VuePoint HD SharpIR” iterative (24 iterations/6 subsets) OSEM resolution-recovery algorithm, correcting for attenuation, scatter, decay, and using a 3 mm Gaussian post-filter, giving a volume of 256 × 256 × 47 voxels of size 0.97 × 0.97 × 3.27mm^3^.

### Data analysis

The PET image data were preprocessed as described earlier [19], using FMRIB's Linear Image Registration Tool (FLIRT) (https://fsl.fmrib.ox.ac.uk/), and volumes of interest (VOIs) were created using FreeSurfer version 6.0.0 (http://surfer.nmr.mgh.harvard.edu/) through automatic segmentation of the individual high-resolution T1-weighted MR images (Discovery MR750, GE Medical Systems) collected in 3D mode (1 mm slice thickness, field of view 25 × 25 cm). The left and right hemisphere segmented gray matter VOIs of the frontal, parietal, temporal and occipital lobes, the cingulate gyrus, the striatum and the gray matter of the cerebellum were used in this study.

Both FE-PE2I- and H_2_O data were collected without arterial blood sampling. Instead, rCBF_R_ was calculated using pharmacokinetic modeling (see below) where, for each tracer, the combined left and right gray matter cerebellum VOIs were used as the reference region. In H_2_O, the measure of rCBF_R_, *F*, was estimated following the double-integration method presented by Koopman et al. [28], but modified to use the cerebellum instead of the whole brain as a fix-point for the perfusion value 0.5 mL × cm^−3^ × min^−1^. A similar measure of rCBF_R_, *R1*, was calculated from the dynamic FE-PE2I data, using SRTM [29, 30]. It is worth noting that the *R1* values are expected to be two times the perfusion values (due to the 0.5 cerebellum fix-point mentioned above). All time points of the dynamic acquisition were used in the modeling. The modeling was performed on VOI level for the time–activity curves of the segmented VOIs.

The same models were also used to create *R1* and *F* rCBF_R_ images. This voxel-level analysis was performed on the dynamic data, smoothed with a kernel of 10 mm. These rCBF_R_ images were used to create correlation images on the voxel level in the following manner:

1. T1 images were segmented into tissues using SPM12, and a brain mask was produced to remove non-brain tissue [36].
2. Skull-stripped T1 images were normalized to Montreal Neurological Institute (MNI) space using FMRIB’s Linear Image Registration Tool (FLIRT) and 12 degrees of freedom (DOF) to achieve a preliminary estimation, followed by a nonlinear registration using FMRIB’s Nonlinear Image Registration Tool (FNIRT) [37].
3. The resulting deformation fields were applied to *R1* and *F* volumes to produce normalized images with 2 mm isotropic voxels.
4. The correlation, *r*, between *R1* and *F* in each voxel was calculated separately for HC and patients using Pearson’s correlation in MATLAB.
5. The resulting volumes were then projected onto cortical surfaces using the Human Connectome Workbench (https://www.humanconnectome.org/software/connectome-workbench) and S1200 data set (https://www.humanconnectome.org/study/hcp-young-adult/document/1200-subjects-data-release).

### Statistics

Statistical analysis and figures were made in SPSS (IBM SPSS Statistics for Windows, Version 25.0. Armonk, NY: IBM Corp.). Prior to any inference test, observations ≥ 2.5 SD above the mean were considered as outliers and were excluded. One patient and one HC fulfilled outlier criteria in the estimation of *F*. There were no outliers in *R1*. Pearson’s correlation analysis was used for assessing the association between relative cerebral perfusion *R1* measured with FE-PE2I and *F* measured with H_2_O in the frontal, parietal, temporal and cingulate cortex as well as in the putamen and caudate nucleus. Average measures for ICC estimates and their 95% confidence intervals were calculated using the “Reliability analysis module” in SPSS based on a two-way mixed-effects model, mean-rating (*k* = 59) using the consistency definition. Bland–Altman plots were made for the regions that fulfilled the condition of normally distributed differences between *R1* and *F* according to the Shapiro–Wilk test (see Additional file 1: Table s3).

Group differences were analyzed using t-tests. For simplicity, correlation analyses, group comparisons and plots of the *r* rCBF_R_ in the respective brain areas were calculated using the averaged left and right hemisphere VOI values, i.e., the mean activity of the respective brain areas. *P* values < 0.05 (two-tailed) were considered statistically significant.

## Results

The *F* and *R1* values, averaged over the left and right hemispheres, stratified by patients and HC, are shown in Table 2.

**Table 2:** rCBF_R_ measured with *F* and *R1* in patients and healthy controls

Region	rCBF_R_	Group	*N*	Mean	SD	SEM
Frontal cortex	*F*	Patients	29	0.37	0.03	0.005
		HC	30	0.38	0.03	0.005
	*R1*	Patients	29	0.93	0.04	0.008
		HC	30	0.93	0.04	0.008
Parietal cortex	*F*	Patients	29	0.35	0.02	0.005
		HC	30	0.37	0.03	0.005
	*R1*	Patients	29	0.89	0.04	0.008
		HC	30	0.92	0.04	0.008
Temporal cortex	*F*	Patients	29	0.36	0.03	0.005
		HC	30	0.38	0.03	0.006
	*R1*	Patients	29	0.86	0.05	0.009
		HC	30	0.87	0.05	0.009
Cingulate cortex	*F*	Patients	29	0.44	0.04	0.008
		HC	30	0.44	0.04	0.008
	*R1*	Patients	29	0.95	0.05	0.009
		HC	30	0.94	0.05	0.010
Occipital cortex	*F*	Patients	29	0.37	0.02	0.005
		HC	30	0.39	0.02	0.004
	*R1*	Patients	29	0.92	0.05	0.009
		HC	30	0.94	0.05	0.009
Putamen	*F*	Patients	29	0.48	0.04	0.008
		HC	30	0.50	0.04	0.008
	*R1*	Patients	29	1.02	0.06	0.011
		HC	30	1.07	0.06	0.012
Caudate	*F*	Patients	29	0.24	0.03	0.006
		HC	30	0.25	0.04	0.007
	*R1*	Patients	29	0.73	0.06	0.012
		HC	30	0.76	0.08	0.014

Values are averaged over the left and right hemisphere. *rCBF*_*R*_ Relative regional cerebral blood flow**,** *HC* Healthy controls*, rCBF*_*R*_ relative regional cerebral blood flow, *F* rCBF_R_ measured with [^15^O]H_2_O PET, *R1* rCBF_R_ measured with [^18^F]FE-PE2I

Across all subjects, the linear correlation between *R1* and *F* in all areas investigated was high, with Pearson’s correlation coefficients ranging between 0.747 and 0.920 in HC and 0.677–0.917 in patients (Table 3).

**Table 3:** Correlations between rCBF_R_ measured by *F* and *R1* by region

Region	Healthy controls (*n* = 30)	Patients (*n* = 29)	All (*n* = 59)
	*r*	*r*	*r*
Frontal cortex	0.820***	0.870***	0.840***
Parietal cortex	0.839***	0.821***	0.847***
Temporal cortex	0.859***	0.907***	0.873***
Occipital cortex	0.747***	0.677***	0.731***
Cingulate cortex	0.908***	0.917***	0.905***
Putamen	0.792***	0.687***	0.766***
Caudate	0.920***	0.816***	0.879***

Regional measures are averaged over the left and right hemispheres. ***p < 0.001. *r*: Pearson correlation coefficient. *rCBF*_*R*_ Relative regional cerebral blood flow. *F* rCBF_R_ measured with [^15^O]H_2_O PET. *R1* rCBF_R_ measured with [^18^F]FE-PE2I

**Fig. 2:**
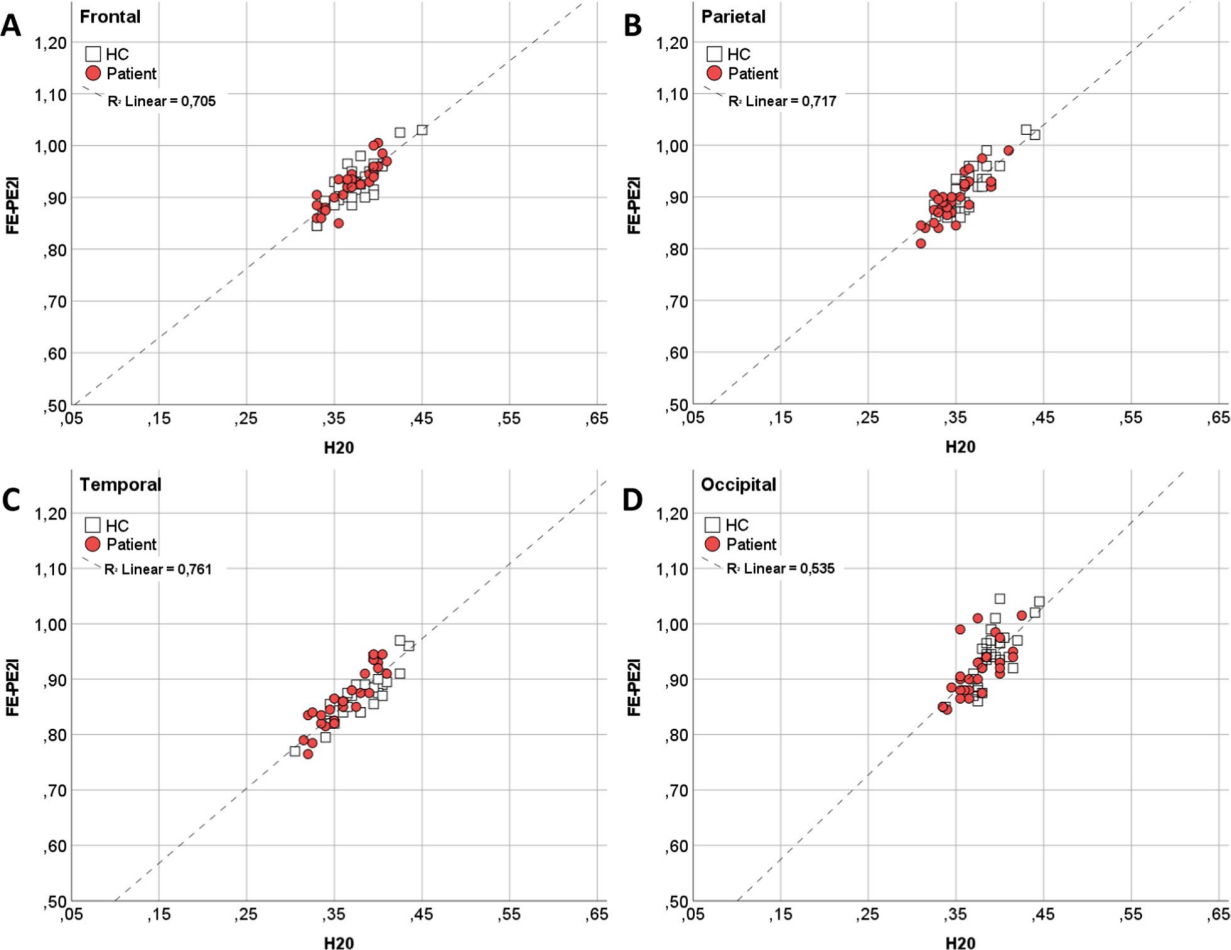
Correlation plots of *R1* (FE-PE2I) and *F* (H_2_O). *Legend* (**A**) Frontal cortex, (**B**) parietal cortex, (**C**) temporal cortex, (**D**) occipital cortex. Values are averaged over the left and right hemispheres

In Fig. 2, the relationships between *R1* and *F* in the frontal, parietal, temporal and occipital cortices are illustrated graphically as plots (correlation plots for the cingulate cortex, caudate and putamen are available in Additional file 2: Fig. s1). For all subjects, *R1* was proportional to *F* but with an *R1*-offset of approximately 0.4; hence, the consistency definition of ICC was used instead of the absolute agreement definition of ICC. The ICC values are presented in Table 4, and Bland–Altman plots are provided in Additional file 3: Fig. s2.

**Table 4:** Intraclass correlation for rCBF_R_ measured by *F* and *R1,* whole sample, *n* = 59

Region	ICC	95% CI
Frontal cortex	0.861***	0.767–0.918
Parietal cortex	0.854***	0.754–0.913
Temporal cortex	0.886***	0.808–0.932
Occipital cortex	0.727***	0.542–0.838
Cingulate cortex	0.943***	0.904–0.966
Putamen	0.832***	0.717–0.900
Caudate	0.838***	0.727–0.904

Regional measures are averaged over the left and right hemispheres. ***p < 0.001

**Fig. 3:**
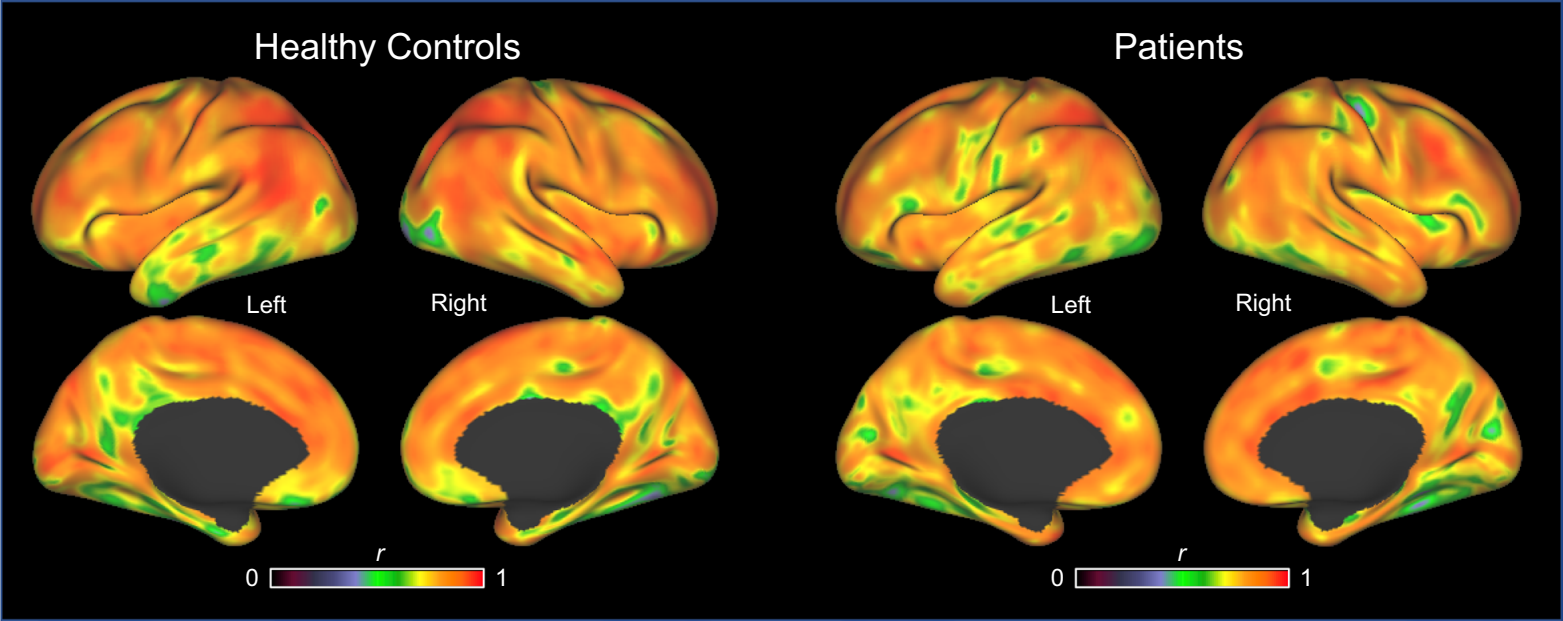
Voxelwise correlations between *R1* and *F* for healthy controls and patients. *Legend* Correlations, *r*, between *R1* and *F* are shown on the cortical surface (https://www.humanconnectome.org/software/connectome-workbench) for healthy controls and patients. High correlations are shown in orange and red color; medium to high correlations are shown in green and yellow

The voxelwise correlations between *R1* and *F* over the hemispheres in healthy controls and patients are illustrated in Fig. 3. The correlations were generally high throughout the cortex, but somewhat lower in the occipital cortex and areas belonging to the brain’s default mode network [38].

On the group level, there was a significant difference between patients and HC in mean rCBF_R_ measured with both *F* and *R1* in the parietal cortex (*p* = 0.004 and 0.020, respectively) and the occipital cortex (*p* = 0.009 and 0.043, respectively) as well as in the temporal lobe measured with *F (p* = 0.024); no significant differences between HC and patients were seen in the other cortical areas (Additional file 4: Table s1).

**Fig. 4:**
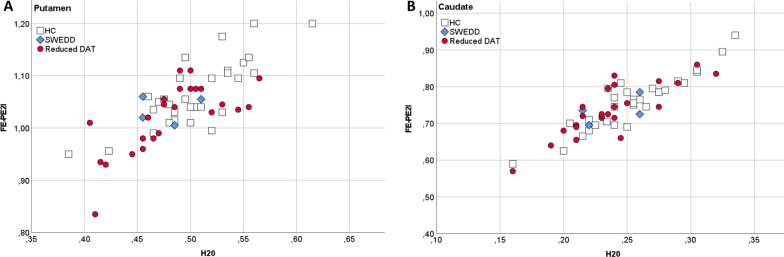
Relative regional cerebral blood flow (rCBF_R_) in relation to striatal DAT activity. *Legend* Plots illustrating the relationship between rCBF_R_ in (**A**) putamen and (**B**) the caudate, measured by *R1* (FE-PE2I) and *F* (H_2_O) and striatal DAT activity. Red circles: patients with pathologically reduced striatal DAT uptake. Blue rhombs: patients without signs of dopaminergic deficit, “SWEDD” (i.e., normal striatal DAT activity). Open squares: healthy controls (HC) with normal DAT activity in the striatum

In Fig. 4, the relation between rCBF_R_ measured in the putamen and caudate with *R1*and *F* is illustrated by plots grouped by HC, patients without signs of dopaminergic deficit (“SWEDDs”) and patients with reduced striatal DAT activity.

No statistical difference was seen in rCBF_R_ (with either *R1* or *F*) in the putamen or the caudate between the SWEDD patients and HC or between the SWEDD patients and the 25 patients with reduced striatal specific binding of FE-PE2I (i.e., reduced DAT activity). There were, however, few observations in these subsamples, and a lack of difference should be interpreted with caution.

In contrast, there was a significant difference in *R1* in the putamen between the group of patients with reduced striatal DAT activity and HC (*p* = 0.012). However, this was not observed in *F (p* = *0.052).* No statistically significant differences were observed in caudate rCBF_R_ measured with *F (p* = 0.089) or *R1* (*p* = 0.236); see Additional file 5: Table s2.

## Discussion

The findings of a strong correlation between *F* and *R1* in cortical and striatal regions are in line with similar studies comparing [^18^F]FDG PET and the outcome of *R1* derived from [^11^C]PE2I PET in 16 patients with parkinsonism [13], the outcome of *R1* derived from [^18^F]FP-Cit PET in 25 PD patients and 16 HC [35], and the outcome of *R1* [^18^F]FP-Cit PET with [^99m^Tc]ECD single photon emission computed tomography (SPECT) in 20 patients with PD [11]. Even though the tracers, the methodology and the number of patients differ between the present and the previously published studies, the pattern of correlating outcome is similar.

As illustrated in Figs. 2 and 3, the correlations between *R1* and *F* were generally high throughout the cortex. Notably, in the occipital cortex, and areas belonging to the brain´s default mode network [38], correlations were slightly lower. The lower correlations in these areas might be due to the function of these regions and the fact that *R1* and *F* were not measured simultaneously. For example, rCBF_R_ in the occipital cortex is influenced by having the eyes open or eyes closed, or by mental imagery. We cannot guarantee that these factors were identical between the two scans. Regarding the medial parietal cortex and temporal lobe areas, both areas belong to the brain’s default mode network and are involved with internally generated thought [39]. Conceivably, regions involved with such processes may also differ in blood flow when comparing *R1* and *F* scans separated in time. Accordingly, this may indicate that neither of the above-mentioned regions would be suitable as reference regions for calculating *R1* or *F*.

The findings of lower rCBF_R_ values in patients compared to HC are also in line with similar findings in previous studies with [^18^F]FDG PET and perfusion SPECT in recent onset, drug naïve and non-demented idiopathic parkinsonism[40–42], strengthening the notion of the usefulness of rCBF_R_ assessment with *R1* in FE-PE2I. The close linear association and high intraclass correlation between estimation of rCBF_R_ with *R1* in FE-PE2I and *F* in H_2_O supports the idea that this technique is viable for differential diagnosis. However, since in our study the majority of the patients had early-stage PD, analyses of potential differences in rCBF_R_ between patients with early and later disease stages or atypical parkinsonian syndromes were not possible. Nonetheless, we see no obvious reason why the method of estimating cortical rCBF_R_ with *R1 *per se would not be purposeful or valid in later-stage PD or in atypical parkinsonism. However, a raise of concern might be the interpretation of rCBF_R_ measured with *R1* in the striate, especially in the putamen, which probably should be taken with caution. According to our findings, a significant difference in rCBF_R_ measured with *R1* (but not *F)* was observed in the putamen between patients with a reduced specific DAT binding of FE-PE2I and HC. Possibly, this might be related to the high DAT affinity and fast kinetics of FE-PE2I, with a rapid binding to the DAT in the striatum. One could hypothesize that the activity in the striatum seen in *R1* of FE-PE2I in the putamen might reflect a mix of perfusion and DAT binding, appearing “normal” in individuals with preserved DAT integrity and “low” in individuals with significant DAT reduction. This may therefore constitute a limitation in patients with suspected MSA-p, who, according to previous studies, are recognized with a significant reduction in rCBF and glucose metabolism in the striatum and cerebellum (as opposed to, for example, PD patients)[43].

Further validation studies including larger numbers of patients with both early- and late-stage PD as well as patients with atypical parkinsonian syndromes or dementia with Lewy bodies are needed to address the usefulness and validity of *R1* rCBF_R_ assessment with FE-PE2I to discriminate between parkinsonian syndromes.

A strength of this study is the prospectively collected image data within a clinical trial and the relatively large number of patients and HC compared to previously published studies on this topic. However, a caveat is the lack of arterial input in PET image acquisition, which did not allow for quantification of the absolute CBF. Nevertheless, in a clinical setting, invasive arterial sampling may be inconvenient or risky, and our results demonstrate that it is possible to estimate rCBF_R_ with H_2_O and FE-PE2I without arterial input. In future studies, a design which allows for comparison between the absolute and relative regional CBF with both methods would be meriting; and such study could also illuminate if absolute perfusion is changed with type of disease and clinical progression.

## Conclusions

This is the first study to show that estimation of the relative regional cerebral blood flow with [^18^F]FE-PE2I PET has a strong association with the relative regional cerebral blood flow measured with [^15^O]H_2_O PET, both in early-stage of parkinsonian disease and in healthy state. This indicates that both the cortical perfusion and the DAT activity can be imaged with one single dynamic [^18^F]FE-PE2I PET scan. This could be particularly valuable in the differential diagnosis of parkinsonian syndromes without necessitating multiple scans and tracers.

## Supplementary Information

**Additional file 1: Table s3**. Test of normally distributed differences between R1 and F. *Legend* *R1* relative regional cerebral blood flow measured with [^18^F]FE-PE2I, *F* relative regional cerebral blood flow measured with [^15^O]H_2_O PET.

**Additional file 2**:** Figure s1**. Correlation plots of R1 (FE-PE2I) and F (H_2_O): cingulate, putamen and caudate. Legend Right: Cingulate cortex, Middle: Putamen, Left: Caudate. Values are averaged over the left and right hemispheres. *HC* Healthy controls (open squares), *Pat* Patients (filled red dots).

**Additional file 3**:** Figure s2**. Bland–Altman plots. Legend Bland–Altman plots illustrating the relationship between the difference between *R1* (FE-PE2I) and *F* (H_2_O) relative regional cerebral blood flow (on the y-axis), and the mean of *R1* and *F* (on the x-axis) in the frontal, parietal and temporal lobes and the cingulate cortex, the putamen and the caudate, respectively. The mean of the difference is indicated by the bold black line. The upper and lower limits of agreement are indicated by dotted red lines. Note Bland–Altman plot was not appropriate for the occipital region due to not normally distributed data (see Supplemental Table s3).

**Additional file 4**:** Table s1**. Difference in rCBF_R_ measured as *F* (H_2_O) and *R1* (FE-PE2I) between patients and healthy controls (T-test results). Legend Measures are averaged left and right hemisphere rCBF_R_ values. rCBF_R_: relative regional cerebral blood flow. *HC* Healthy controls; *rCBF*_*R*_ relative regional cerebral blood flow. *F* relative regional cerebral blood flow measured with [^15^O]H_2_O PET. *R1* relative regional cerebral blood flow measured with [^18^F]FE-PE2I.

**Additional file 5**: **Table s2**. Cerebral flow in the striatum. Healthy controls vs. patients with reduced specific DAT activity in the striatum. Legend Measures are averaged left and right hemisphere rCBF_R_ values. *P* values are two-sided. *rCBF*_*R*_ relative regional cerebral blood flow. *F* relative regional cerebral blood flow measured with [^15^O]H_2_O PET. *R1* relative regional cerebral blood flow measured with [^18^F]FE-PE2I.

## Data Availability

The datasets used and/or analyzed during the current study are available from the corresponding author on reasonable request.

## Abbreviations

DAT: Dopamine transporter
rCBF: Regional cerebral blood flow
rCBF_R_: Relative regional cerebral blood flow
PET: Positron emission tomography
FE-PE2I: [^18^F]FE-PE2I PET
H_2_O: [^15^O]H2O PET
ICC: Intraclass correlation
*R1*: rCBF_R_ measured with [^18^F]FE-PE2I
*F*: rCBF_R_ measured with [^15^O]H_2_O
PD: Parkinson’s disease
SRTM: Simplified reference tissue model
HC: Healthy controls
SD: Standard deviations
SEM: Standard error of the mean
Non-IPS: Non-degenerative Parkinsonian condition
SWEDD: Subjects without evidence of dopaminergic deficit
CT: Computed tomography
MR: Magnetic resonance
VOIs: Volumes of interest
SPECT: Single photon emission computed tomography
