# Supplementary material for: Validation of dynamic [18F]FE-PE2I PET for estimation of relative regional cerebral blood flow: a comparison with [15O]H2O PET
Source: EJNMMI Res. 2022 Nov 17;12:72. doi: 10.1186/s13550-022-00941-8 (PMC9672223; doi:10.1186/s13550-022-00941-8)
Supplement: Supplementary file 1 — Additional file 1: Table s3. Test of normally distributed differences between R1 and F. Legend R1 relative regional cerebral blood flow measured with [18F]FE-PE2I, F relative regional cerebral blood flow measured with [15O]H2O PET. [file 13550_2022_941_MOESM1_ESM.docx]

**Table s3: Test of normally distributed differences between *R1* and *F***

| **Region of measured difference (*R1*-*F*)** | **Shapiro-Wilk test** | | |
| --- | --- | --- | --- |
|  | **W** | **df** | ***p*** |
| Frontal lobe | 0.973 | 59 | 0.213 |
| Parietal lobe | 0.984 | 59 | 0.618 |
| Temporal lobe | 0.966 | 59 | 0.097 |
| Occipital lobe | 0.952 | 59 | *0.020* |
| Cingulate cortex | 0.963 | 59 | 0.071 |
| Putamen | 0.983 | 59 | 0.558 |
| Caudate | 0.992 | 59 | 0.964 |

*R1*: relative regional cerebral blood flow measured with [^18^F]FE-PE2I
*F*: relative regional cerebral blood flow measured with [^15^O]H_2_O PET
