# Supplementary figures and images for: Validation of dynamic [18F]FE-PE2I PET for estimation of relative regional cerebral blood flow: a comparison with [15O]H2O PET
Source: EJNMMI Res. 2022 Nov 17;12:72. doi: 10.1186/s13550-022-00941-8 (PMC9672223; doi:10.1186/s13550-022-00941-8)

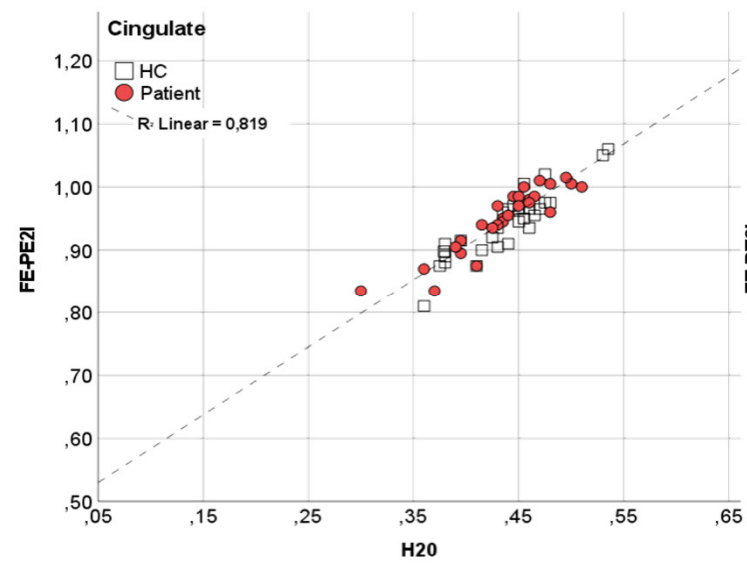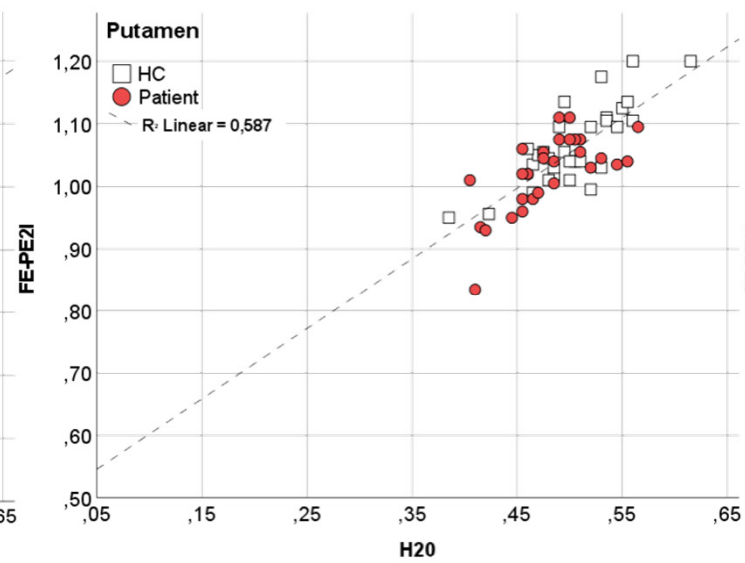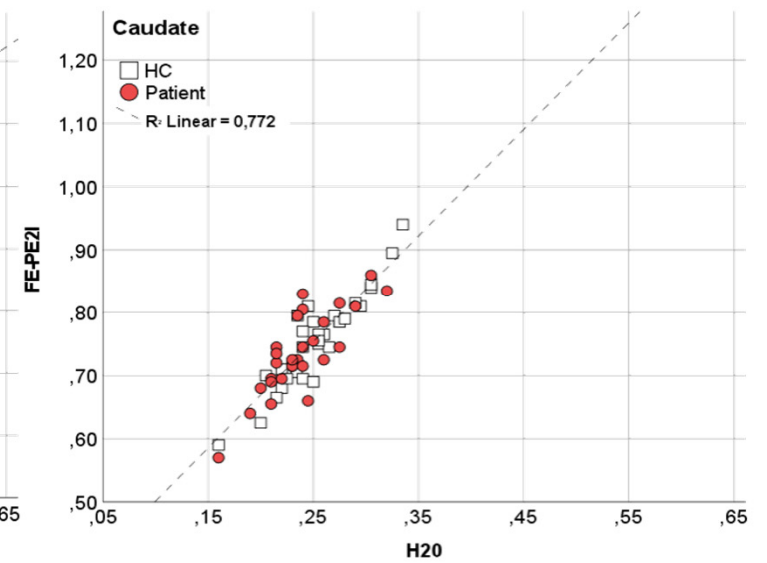

Supplement: Supplementary file 2 — Additional file 2: Figure s1. Correlation plots of R1 (FE-PE2I) and F (H2O): cingulate, putamen and caudate. Legend Right: Cingulate cortex, Middle: Putamen, Left: Caudate. Values are averaged over the left and right hemispheres. HC Healthy controls (open squares), Pat Patients (filled red dots). [file 13550_2022_941_MOESM2_ESM.pdf]

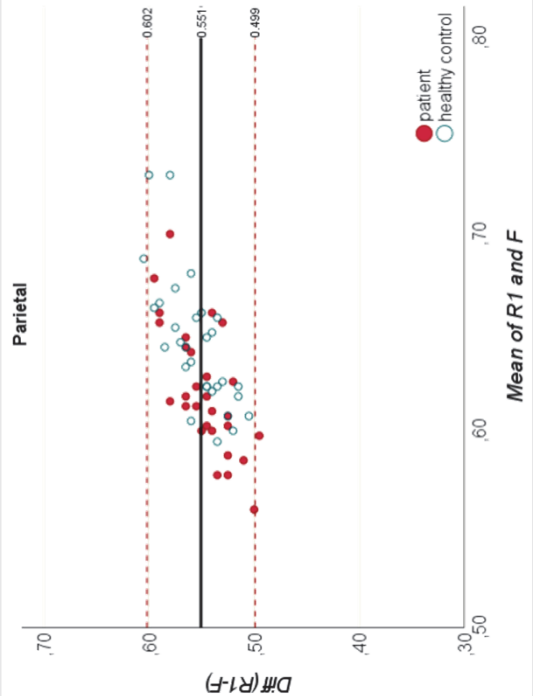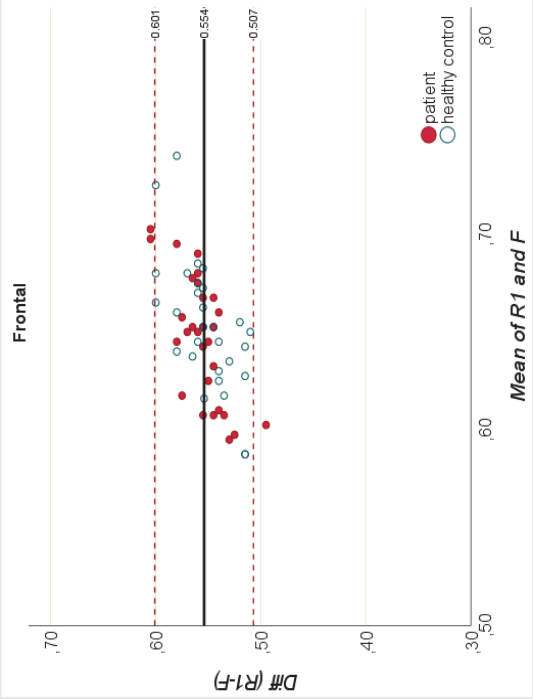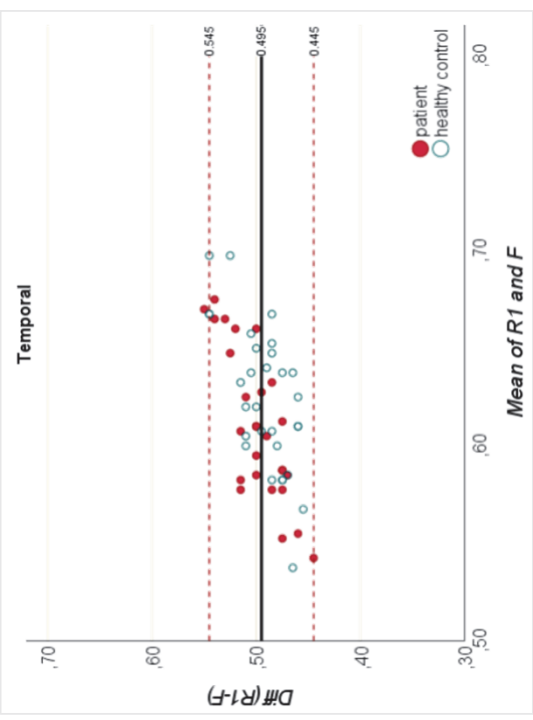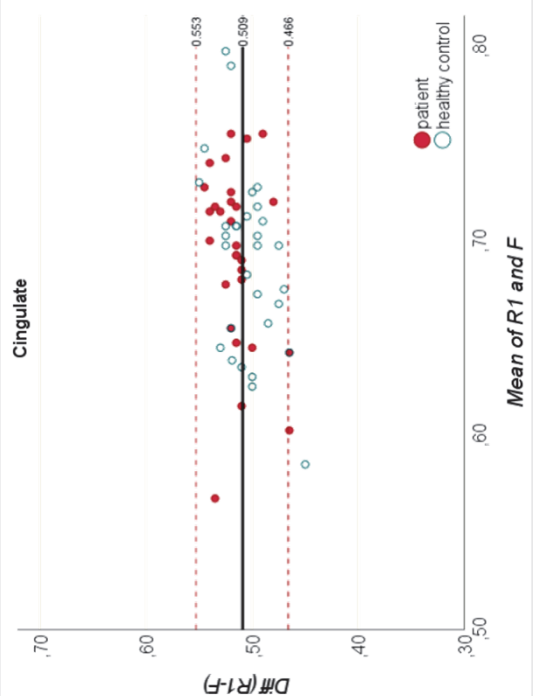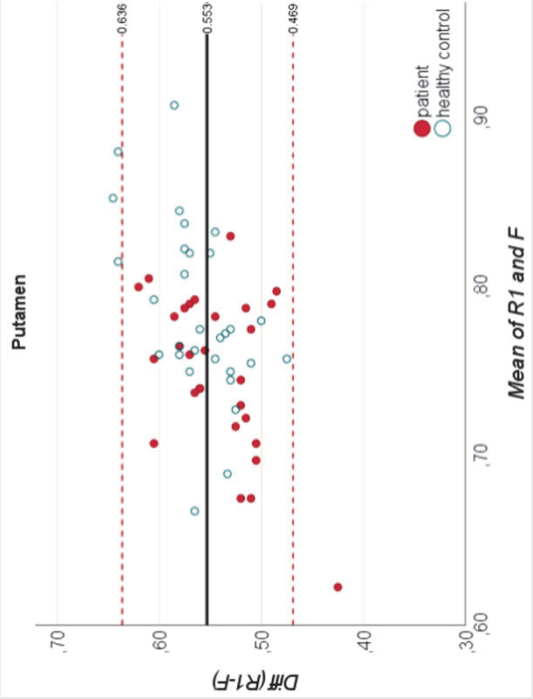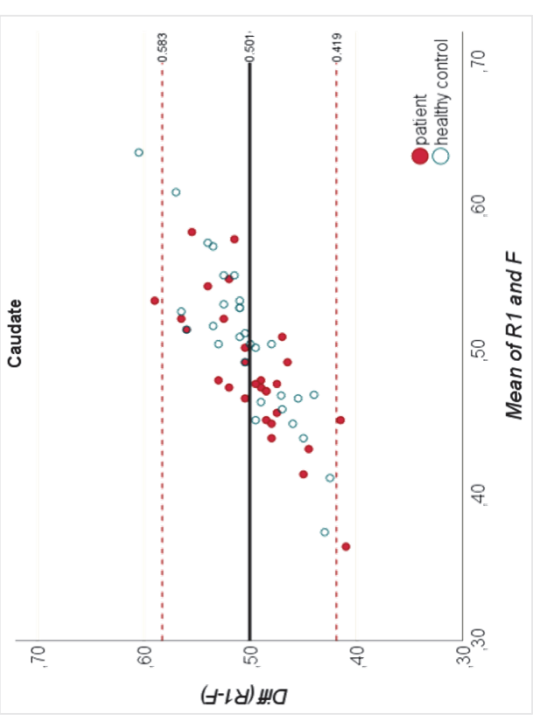

Supplement: Supplementary file 3 — Additional file 3: Figure s2. Bland–Altman plots. Legend Bland–Altman plots illustrating the relationship between the difference between R1 (FE-PE2I) and F (H2O) relative regional cerebral blood flow (on the y-axis), and the mean of R1 and F (on the x-axis) in the frontal, parietal and temporal lobes and the cingulate cortex, the putamen and the caudate, respectively. The mean of the difference is indicated by the bold black line. The upper and lower limits of agreement are indicated by dotted red lines. Note Bland–Altman plot was not appropriate for the occipital region due to not normally distributed data (see Supplemental Table s3). [file 13550_2022_941_MOESM3_ESM.pdf]
