# Supplementary material for: Validation of dynamic [18F]FE-PE2I PET for estimation of relative regional cerebral blood flow: a comparison with [15O]H2O PET
Source: EJNMMI Res. 2022 Nov 17;12:72. doi: 10.1186/s13550-022-00941-8 (PMC9672223; doi:10.1186/s13550-022-00941-8)
Supplement: Supplementary file 4 — Additional file 4: Table s1. Difference in rCBFR measured as F (H2O) and R1 (FE-PE2I) between patients and healthy controls (T-test results). Legend Measures are averaged left and right hemisphere rCBFR values. rCBFR: relative regional cerebral blood flow. HC Healthy controls; rCBFR relative regional cerebral blood flow. F relative regional cerebral blood flow measured with [15O]H2O PET. R1 relative regional cerebral blood flow measured with [18F]FE-PE2I. [file 13550_2022_941_MOESM4_ESM.docx]

**Supplementary material, Tables**

**Table s1: Difference in rCBF_R_ measured as *F* (H_2_O) and *R1* (FE-PE2I) between patients and healthy controls (T-test results)**

| Region | Measure | df | *p*  (2-tailed) | Mean Difference | Std. Error of Difference | 95% CI of the diff. | |
| --- | --- | --- | --- | --- | --- | --- | --- |
|  |  |  |  |  |  | Lower | Upper |
| Frontal cortex | *F* | 57 | 0.231 | -0.008 | 0.007 | -0.022 | 0.005 |
|  | *R1* | 57 | 0.696 | -0.004 | 0.011 | -0.026 | 0.017 |
| Parietal cortex | *F* | 57 | ***0.004*** | -0.020 | 0.007 | -0.033 | -0.007 |
|  | *R1* | 57 | ***0.020*** | -0.027 | 0.011 | -0.050 | -0.004 |
| Temporal Cortex | *F* | 57 | ***0.024*** | -0.018 | 0.008 | -0.033 | -0.002 |
|  | *R1* | 57 | 0.333 | -0.012 | 0.012 | -0.037 | 0.013 |
| Cingulate cortex | *F* | 57 | 0.866 | -0.002 | 0.011 | -0.025 | 0.021 |
|  | *R1* | 57 | 0.505 | 0.009 | 0.014 | -0.018 | 0.036 |
| Occipital cortex | *F* | 57 | ***0.009*** | -0.016 | 0.006 | -0.028 | -0.004 |
|  | *R1* | 57 | ***0.043*** | -0.026 | 0.013 | -0.052 | 0.001 |

Measures are averaged left and right hemisphere rCBF_R_ values

rCBF_R_: relative regional cerebral blood flow. HC: Healthy controls; rCBF_R_: relative regional cerebral blood flow. *F*: relative regional cerebral blood flow measured with [^15^O]H_2_O PET. *R1*: relative regional cerebral blood flow measured with [^18^F]FE-PE2I
