# Supplementary material for: Validation of dynamic [18F]FE-PE2I PET for estimation of relative regional cerebral blood flow: a comparison with [15O]H2O PET
Source: EJNMMI Res. 2022 Nov 17;12:72. doi: 10.1186/s13550-022-00941-8 (PMC9672223; doi:10.1186/s13550-022-00941-8)
Supplement: Supplementary file 5 — Additional file 5: Table s2. Cerebral flow in the striatum. Healthy controls vs. patients with reduced specific DAT activity in the striatum. Legend Measures are averaged left and right hemisphere rCBFR values. P values are two-sided. rCBFR relative regional cerebral blood flow. F relative regional cerebral blood flow measured with [15O]H2O PET. R1 relative regional cerebral blood flow measured with [18F]FE-PE2I. [file 13550_2022_941_MOESM5_ESM.docx]

**Table s2: Cerebral flow in the striatum. Healthy controls vs. patients with reduced specific DAT activity in the striatum**

| **rCBF_R_** | **Category** | **N** | **Mean** | **SD** | **SEM** | ***P*** |
| --- | --- | --- | --- | --- | --- | --- |
| Putamen *F* | Patients with reduced DAT | 25 | 0.48 | 0.044 | 0.009 | 0.052 |
|  | Healthy controls | 30 | 0.50 | 0.045 | 0.008 |  |
| Caudate *F* | Patients with reduced DAT | 25 | 0.24 | 0.036 | 0.007 | 0.089 |
|  | Healthy controls | 30 | 0.25 | 0.038 | 0.007 |  |
| Putamen *R1* | Patients with reduced DAT | 25 | 1.02 | 0.064 | 0.013 | *0.012* |
|  | Healthy controls | 30 | 1.07 | 0.064 | 0.012 |  |
| Caudate *R1* | Patients with reduced DAT | 25 | 0.73 | 0.069 | 0.014 | 0.236 |
|  | Healthy controls | 30 | 0.76 | 0.075 | 0.014 |  |

Measures are averaged left and right hemisphere rCBF_R_ values. *P* values are two-sided

*rCBF_R_*: relative regional cerebral blood flow. *F*: relative regional cerebral blood flow measured with [^15^O]H_2_O PET. *R1*: relative regional cerebral blood flow measured with [^18^F]FE-PE2I
